# Machine learning model identifies tibial anatomical variables as potential risk factors for anterior cruciate ligament injury

**DOI:** 10.1002/ksa.70322

**Published:** 2026-02-06

**Authors:** Cheng‐Hao Kao, Javad Hashemi, James Slauterbeck, Naveen Chandrashekar

**Affiliations:** ^1^ Department of Mechanical and Mechatronics Engineering University of Waterloo Waterloo Ontario Canada; ^2^ Department of Ocean and Mechanical Engineering & Department of Biomedical Engineering Florida Atlantic University Boca Raton Florida USA; ^3^ UNC Health Lumberton North Carolina USA

**Keywords:** anterior cruciate ligament injury, feature importance, machine learning, risk factor, tibial anatomical feature

## Abstract

**Purpose:**

The tibial slope is a well‐known risk factor for anterior cruciate ligament (ACL) injury. As machine learning continues to progress, it has become an increasingly explored tool for clinical screening and risk factor analysis. This study aims to develop and validate a prognostic machine learning model to predict the outcome of ACL injury from tibial anatomic parameters and identify the most predictive features.

**Methods:**

A pre‐published dataset of coronal, medial and lateral tibial slopes and medial tibial depth was constructed using magnetic resonance imaging scans taken from 104 subjects (44 males: 22 injured, 22 uninjured; 60 females: 27 injured, 33 uninjured). The dataset was split into train‐validation and test sets to ensure robust model evaluation. AutoGluon‐enabled machine learning models, including XGBoost, LightGBM, CatBoost, TabPFN, TabM, TabICL, MITRA and their weighted ensembles were trained and tuned with respect to the F2‐score across ten different random seeds. Two instances of the best‐performing model were developed: a *default tested model* (weighted ensemble from the default seed of 42) and a *full‐dataset model* (weighted ensemble retrained on the entire dataset). Global SHapley Additive exPlanations analysis was used to elucidate the most predictive features, and local SHapley Additive exPlanations analysis to provide interpretability for individual predictions.

**Results:**

The *default tested model* achieved a 73.60% validation F2‐score. On the test set, it demonstrated a 95.44% test balanced accuracy, 95.24% F1‐score, 98.04% F2‐score, 100% ROC AUC, 90.91% precision and 100% recall. The *full‐dataset model* achieved an 81.30% validation F2‐score. The relative importance of tibial anatomical features were identified.

**Conclusions:**

Overall, the study presented two prognostic models with moderately high predictive power to identify subjects with high likelihood of ACL injury. Decreased medial tibial depth along with increased medial and lateral tibial slopes were reported as top predictors for ACL injury. These models can potentially be integrated into clinical practice to assist clinicians in predicting the likelihood of ACL injury, but require external validation.

**Level of Evidence:**

Level III, case‐control study.

**Abbreviations**
ACL: anterior cruciate ligament
ACLR: anterior cruciate ligament reconstruction
CTS: coronal tibial slope
DNN: deep neural networks
GBT: gradient boosted tree‐based models
L1: layer 1
L2: layer 2
LTS: lateral tibial slope
ML: machine learning
MTD: medial tibial depth
MTS: medial tibial slope
SHAP: SHapley Additive exPlanations

## INTRODUCTION

It is well known that there are several risk factors that predispose athletes towards anterior cruciate ligament (ACL) injury. Researchers have tried to identify athletes who are at high risk of ACL injury using movement patterns, especially landing from a jump, as a screening tool [13, 25, 31]. Researchers have also tried to identify anatomic risk factors such as tibial slope, femoral notch width and meniscal geometry [3, 6, 16, 41, 42, 48]. These approaches have provided foundational insights into anatomical, biomechanical and clinical factors that contribute to the risk of ACL injury and have helped shape current understanding of ACL injury mechanisms and risk profiles.

Recently, machine learning (ML) techniques have emerged as a promising complement, leveraging classical or deep learning models to achieve high diagnostic and prognostic accuracies by learning patterns from existing datasets [2, 21, 24, 39]. Carefully designed ML models are less prone to human errors, which makes them a reliable option for assisting or replacing a medical professional's judgment [4, 7]. A growing body of literature has demonstrated the capabilities of ML‐based approaches in ACL injury outcome predictions [12, 38].

Among anatomical features, tibial slope and tibial depth have been increasingly recognised as major risk factors for ACL injury and ACLR (ACL reconstruction) rupture [3, 35, 52]. Consequently, these measurements have been included in a growing number of ML prognostic frameworks. Some studies have conducted rigorous regression studies focusing on the different components of tibial slope and tibial depth, but were primarily designed to model linear associations and therefore do not explicitly higher‐order inter‐feature interactions [16, 23]. In contrast, other studies have employed state‐of‐the‐art ML models for ACL injury prediction but did not distinguish among the different components of tibial slope, thereby limiting the granularity of anatomical interpretation [51, 53]. One exception is the study by Tamimi et al., which employed Gaussian naïve Bayes models and utilised various tibial slope as well as meniscal height measurements [44]. However, the conditional independence assumption inherent to this approach restricts the model's ability to capture inter‐feature dependencies, while the absence of feature importance analysis further limits the interpretability of model predictions. As a result, the role of the different components of tibial slope and depth remains insufficiently explored using state‐of‐the‐art, explainable ML techniques.

To address this gap, the current study adopts state‐of‐the‐art, explainable ML models capable of capturing inter‐feature interactions, with a specific focus on the different tibial slopes and tibial depth to provide a more comprehensive analysis of potential ACL injury risk factors among tibial anatomical measurements. It is hypothesised that the resulting models trained with detailed tibial slope and depth measurements will demonstrate strong predictive performance and provide interpretable insights into the relative contributions of individual tibial anatomical features to ACL injury outcomes.

## METHODS

### MRI measurement

**Figure 1:**
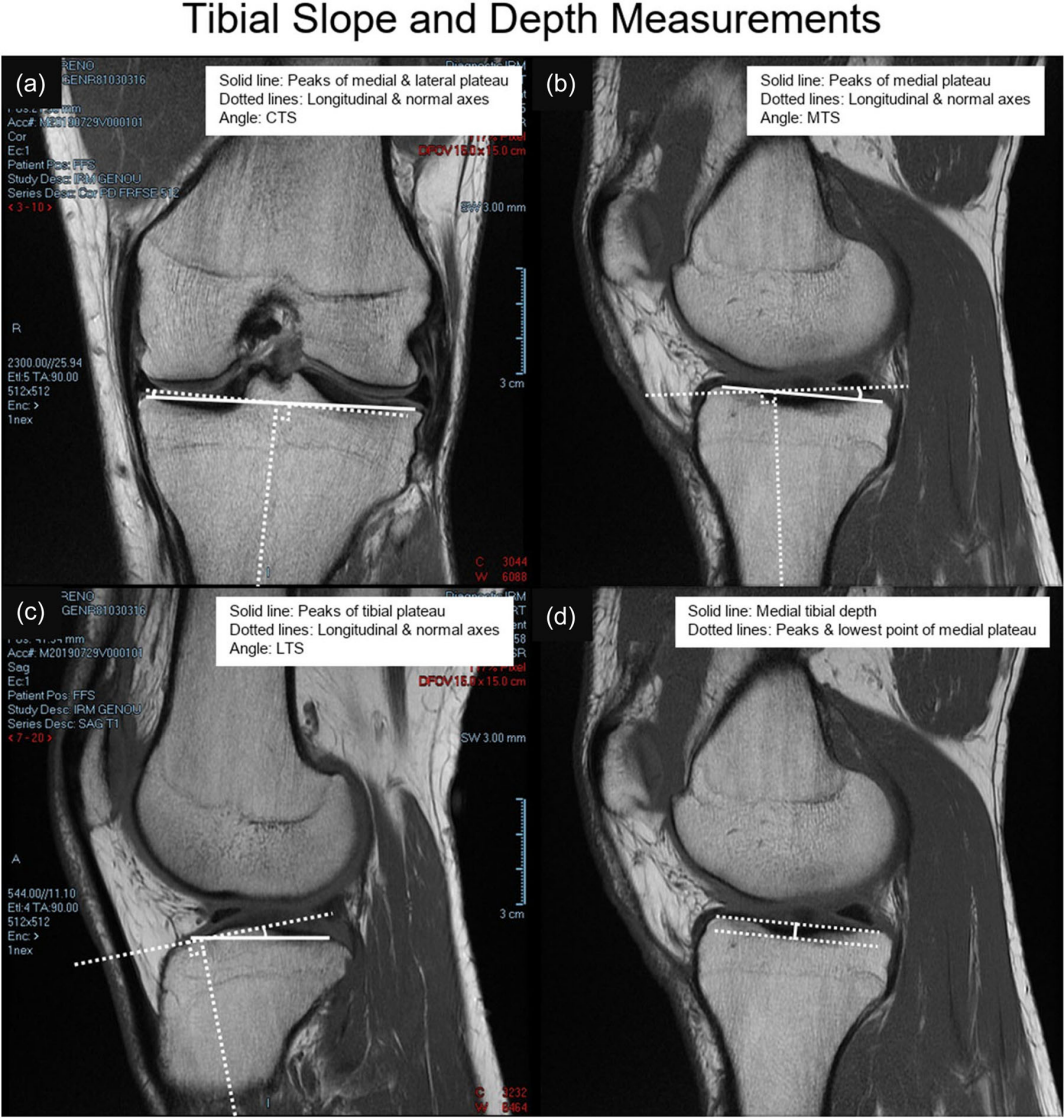
Illustrative examples of tibial anatomical measurements taken by Hashemi et al. [15]. These measurements include coronal tibial slope (CTS) (a), medial tibial slope (MTS) (b), lateral tibial slope (LTS) (c) and medial tibial depth (MTD) (d).

The dataset used in the study was originally collected by Hashemi et al. [15, 16]. It consisted of MRI scans from 47 ACL‐injured and 55 uninjured participants. Anatomical features such as coronal tibial slope (CTS), medial tibial slope (MTS), lateral tibial slope (LTS) and medial tibial depth (MTD) were extracted from the scans, as shown in Figure 1. Details regarding the exact procedures for determining each tibial slope and tibial depth can be found in [15]. They are currently one of the two most commonly adopted measurement techniques, the other being the *Hudek* method, but have yet to be universally accepted by all studies [52].

### Data pre‐processing and splitting

Among the data collected, CTS, MTS, LTS, MTD and sex were identified as features whereas injury was designated as the label. The features were a mix of quantitative (CTS, MTS, LTS and MTD) and categorical (sex) variables, while the injury label was also categorical. The dataset was divided into a train‐validation set (80%) and a test set (20%) using stratified sampling based on the injury label to ensure that the class distribution was preserved in both subsets. Since the classes were roughly balanced, imbalanced‐learning techniques were deemed unnecessary.

The detailed mean and standard deviation statistics for these groups and subsets were computed and are presented in the *Results* section (Tables 1, 2, 3). A detectable‐effect power analysis for logistic regression was conducted using a sample size of 104, significance level of 0.05, and power of 0.8, and ACL injury prevalence of roughly 0.45.

**Table 1:** Mean and standard deviation statistics computed from the train‐validation set and the test set for the default random seed of 42.

	Train‐validation set	Test set
Sex		
Male	36	8
Female	47	13
CTS (⁰)	2.9±2.2	3.2 ± 1.8
MTS (⁰)	5.7 ± 3.1	5.9±3.2
LTS (⁰)	7.2 ± 2.8	7.0±3.6
MTD (mm)	2.4 ± 1.0	2.4 ± 1.1

*Note*: They are expressed in the format of mean ± standard deviation.

Abbreviations: CTS, coronal tibial slope; LTS, lateral tibial slope; MTD, medial tibial depth; MTS, medial tibial slope.

**Table 2:** Mean and standard deviation statistics for the injured and uninjured groups, computed from the entire dataset.

	Uninjured	Injured
Sex		
Male	22	22
Female	33	27
CTS (⁰)	3.0 ± 1.9	2.9 ± 2.3
MTS (⁰)	5.0 ± 3.2	6.5 ± 3.2
LTS (⁰)	6.4 ± 3.0	7.9 ± 2.8
MTD (mm)	2.9 ± 0.9	1.9 ± 1.0

*Note*: They are expressed in the format of mean ± standard deviation.

Abbreviations: CTS, coronal tibial slope; LTS, lateral tibial slope; MTD, medial tibial depth; MTS, medial tibial slope.

**Table 3:** Mean and standard deviation statistics for the injured and uninjured groups of male and female participants, computed from the entire dataset.

	Uninjured male	Injured male	Uninjured female	Injured female
Group Size	22	22	33	27
CTS (⁰)	3.5 ± 1.9	3.3 ± 2.5	2.6 ± 1.9	2.6 ± 2.1
MTS (⁰)	3.7 ± 3.1	6.1 ± 2.7	5.9 ± 3.0	6.9 ± 3.6
LTS (⁰)	5.4 ± 2.8	7.2 ± 2.7	7.1 ± 2.9	8.4 ± 2.8
MTD (mm)	3.1 ± 1.0	2.0 ± 1.0	2.7 ± 0.8	1.9 ± 1.0

*Note*: They are expressed in the format of mean ± standard deviation.

Abbreviations: CTS, coronal tibial slope; LTS, lateral tibial slope; MTD, medial tibial depth; MTS, medial tibial slope.

### ML pipeline

The experiments conducted in this study were carried out within a Python 3.11.13 environment, leveraging the ML capabilities provided by AutoGluon 1.4.0. AutoGluon‐Tabular (referred to as AutoGluon for conciseness) is an AutoML framework designed by Erickson et al. to handle tabular data tasks [10]. Its *extreme* preset, recently inspired by TabArena [11] and added in version 1.4.0., was specifically designed to tackle small tabular datasets.

This study evaluated multiple ML models, including gradient boosted tree‐based models (GBTs) and modern deep neural networks (DNNs) designed for small tabular datasets. The GBTs, including XGBoost, LightGBM and CatBoost, were chosen for their well‐established performance on tabular datasets, as highlighted in [14, 22]. Modern specialised DNNs, such as TabPFN, TabM, TabICL and Mitra, were included to represent the state‐of‐the‐art approaches in this domain [30]. Instead of viewing GBTs and DNNs as competing methodologies, this work followed the recommendations made by Airlangga and Liu [1], utilising AutoGluon to ensemble both model families such that their strengths can be combined. This was made possible by k‐fold bagging [32] and multi‐layer stacking [10], techniques that aggregate models through a weighted voting strategy to improve robustness and reduce the risk of overfitting.

To ensure robustness and avoid dependence on arbitrary data splits, models were trained across multiple random seeds. The F2‐score, which favours recall/sensitivity over precision, was used as the primary optimisation metric, reflecting clinical preference to avoid false‐negative predictions. Other supplementary metrics, including balanced accuracy, F1‐score, ROC AUC, precision and recall were reported for model evaluation to ensure overall reliability. Once optimised, the best‐performing model trained on the default seed of 42 was selected, referred to as the *tested default model*. Another version of the same model, referred to as the *full‐dataset model*, was subsequently retrained on the entire dataset to explore potential performance gains in clinical deployment, though the model cannot be independently evaluated.

### Model explainability

Model explainability was examined using SHapley Additive exPlanations (SHAP) analysis [29]. Global SHAP explanations were used to identify which features the model relied on most frequently across all predictions, while local SHAP explanations were used to highlight the influence features have on specific subject‐level predictions. SHAP was used to interpret model behaviour for elucidating potential risk factors for ACL injuries, rather than biomechanically establishing causal relationships.

## RESULTS

### Data characteristics

Descriptive statistics for the different groups of participants involved in the study are presented in Tables 1, 2, 3. More detailed comparison between different groups can be found in [16]. Based on the assumed parameters for power analysis (α=0.05,β=0.8,ACLinjuryprevalence≈0.45,n=104), the study is adequately powered to reliably detect an odds ratio of approximately 1.7 or greater.

### Model performance

Shown in Table 4 is a leaderboard of various models trained across 10 different random seeds by the AutoGluon pipeline. For conciseness, the table presents only the top‐performing model from each classifier type, selected based on their validation F2‐scores. The weighted L2 ensemble achieves the highest validation performance and was therefore selected as the best model for subsequent analysis.

**Table 4:** Aggregated results of all models trained and tuned by AutoGluon across ten different random seeds.

Classifier	Validation score (%)	Test balanced accuracy (%)	Test F1‐score (%)	Test F2‐score (%)	Test ROC AUC (%)	Test precision (%)	Test recall (%)
WeightedEnsemble_L2	78.62 ± 5.23	73.41 ± 9.09	72.43 ± 10.04	73.76 ± 12.76	80.73 ± 9.65	71.61 ± 9.87	75.00 ± 15.09
TabM_r49_BAG_L1	77.90 ± 5.24	72.45 ± 9.02	71.42 ± 10.10	72.79 ± 12.81	80.09 ± 9.45	70.27 ± 8.65	74.00 ± 15.06
LightGBM_r33_BAG_L1	65.89 ± 8.53	70.73 ± 10.26	64.16 ± 14.85	58.86 ± 16.27	78.82 ± 10.86	78.15 ± 15.21	56.00 ± 17.13
XGBoost_r40_BAG_L1	64.72 ± 5.94	70.23 ± 9.40	63.03 ± 15.08	57.75 ± 17.13	79.45 ± 12.05	77.44 ± 12.71	55.00 ± 18.41
Mitra_BAG_L1	64.31 ± 8.10	72.95 ± 10.26	69.22 ± 12.81	66.48 ± 15.00	79.45 ± 10.53	76.18 ± 12.01	65.00 ± 16.50
CatBoost_r91_BAG_L1	63.86 ± 6.58	69.68 ± 9.61	66.34 ± 11.78	64.21 ± 12.64	78.27 ± 12.92	71.08 ± 11.97	63.00 ± 13.37
TabICL_BAG_L1	60.27 ± 7.21	76.32 ± 8.69	73.33 ± 11.25	70.58 ± 12.71	77.82 ± 10.00	79.32 ± 9.72	69.00 ± 13.70
TabPFNv2_r143_BAG_L1	59.65 ± 6.90	67.23 ± 10.92	62.38 ± 14.34	60.13 ± 16.97	77.36 ± 11.10	68.99 ± 11.16	59.00 ± 18.53

*Note*: The models are ranked in order of validation performance. The suffix *_BAG_L1* indicates a Layer‐1 bag model in the multi‐layer stacking architecture, while *WeightedEnsemble_L2* indicates an Layer‐2 weighted combination of models from Layer 1. Numerical values in the table are presented in the format of mean ± standard deviation.

Abbreviations: AUC, area under curve; ROC, receiver operating characteristic.

Despite moderately high standard deviations in the test metrics according to Table 4, the weighted L2 ensemble consistently produced a test F2‐score within the ±7% range of their validation counterparts. Having found the best model type from the aggregated results, an L2 weighted ensemble model trained on the default random seed of 42 is presented. This model, referred to as the *default tested model*, is a weighted combination of *TabM_r49_BAG_L1* (weight = 0.75) and *LightGBM_r11_BAG_L1* (weight = 0.25). Its validation and test performance can be found in Table 5. The test scores achieved by this model are notably higher than the corresponding validation metrics. The test F2‐score, in particular, is 24.44% higher than the validation F2‐score (and 24.28% higher than the average Test F2‐score), making this seed one of the only three that observe an increase in performance when experiencing a distributional shift from the train‐validation set to the test set.

**Table 5:** Validation and test performance achieved by the *default tested model* and *full‐dataset model*.

Model	Validation score (%)	Test Balanced accuracy (%)	Test F1‐score (%)	Test F2‐score (%)	Test ROC AUC (%)	Test precision (%)	Test recall (%)
Default tested model	73.60	95.45	95.24	98.04	100.00	90.91	100.00
Full‐dataset model	81. 30	NA	NA	NA	NA	NA	NA

Abbreviations: AUC, area under curve; ROC, receiver operating characteristic.

By merging the original test set into the train‐validation set, another version of the weighted L2 ensemble was obtained. This version of the model, referred to as the *full‐dataset model*, is a combination of *LightGBM_r33_BAG_L1* (weight ≈ 0.43), *TabM_r49_BAG_L1* (weight ≈ 0.43), and *XGBoost_r40_BAG_L1* (weight ≈ 0.14). Since the *full‐dataset model* is trained and tuned on the entirety of the original dataset, it cannot be independently evaluated. Consequently, only the validation performance is presented in Table 5.

### Confidence and feature importance

**Figure 2:**
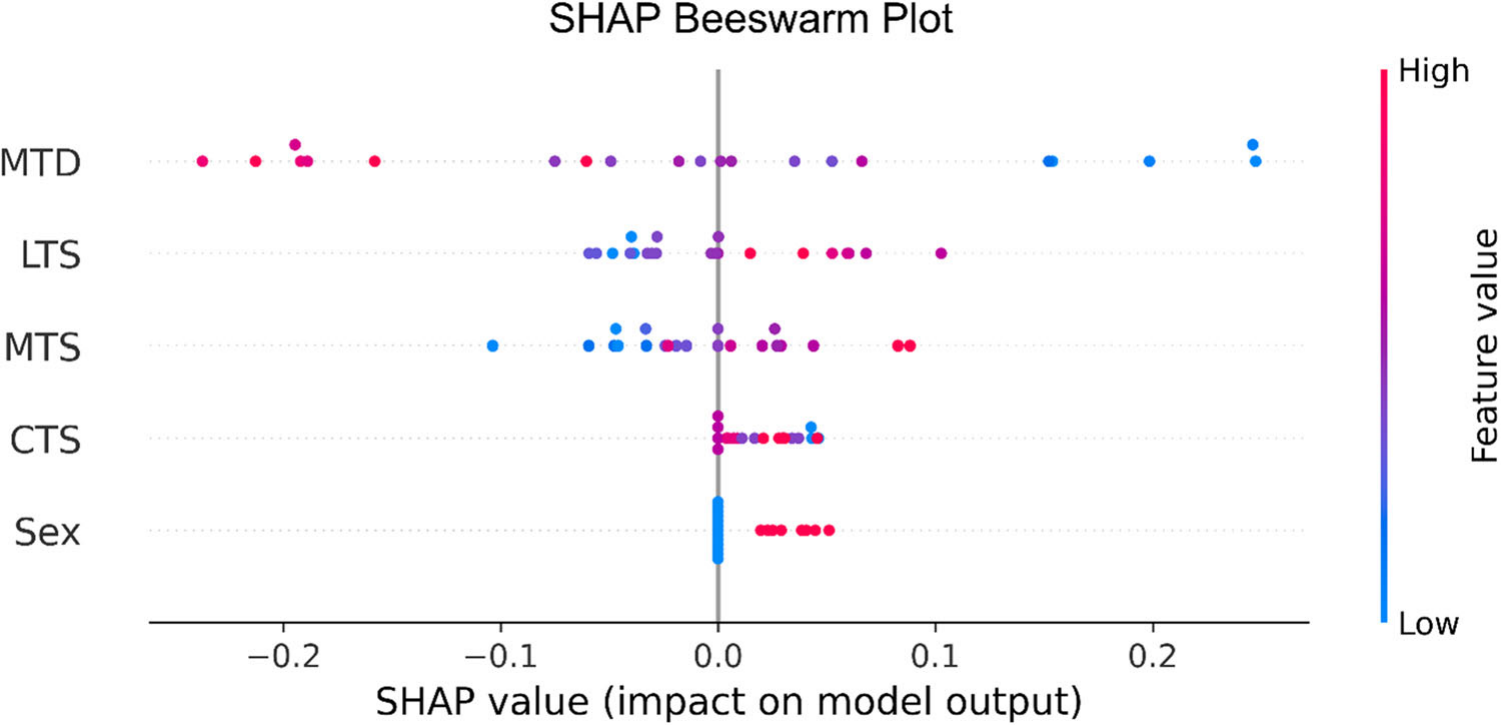
The SHapley Additive exPlanations (SHAP) beeswarm plot showcases the SHAP values for different tibial anatomical features of the *default tested model*. The y‐axis ranks the importance of tibial anatomical features based on their mean absolute SHAP values. The *x*‐axis represents the SHAP value, which quantifies each feature's contribution to the model's outputs. Positive SHAP values contribute toward predictions of higher likelihood of anterior cruciate ligament (ACL) injury, whereas negative SHAP values contribute toward predictions of lower likelihood of ACL injury. Red represents high value of the feature, blue represents low value of the feature. CTS, coronal tibial slope; LTS, lateral tibial slope; MTD, medial tibial depth; MTS, medial tibial slope.

With the presence of a test set, global feature importance analysis can be conducted on *the default tested model* via SHAP analysis. The results are presented in a SHAP beeswarm plot in Figure 2. A SHAP beeswarm plot is a visual summary of how each feature in a predictive model influences the model's output across all subjects. Every dot in the plot represents a single subject's SHAP value for a specific feature. For an individual patient, a SHAP value indicates how much a specific feature increased or decreased the model's predicted outcome compared with an average prediction. It can be seen from Figure 2 that the feature importance is ranked in descending order: MTD, LTS, MTS, CTS and sex. The red and blue data points are well‐separated and symmetrical about the centre axis (SHAP value = 0) for the top three features, with red mostly clustered on the negative side for MTD and on the positive side for LTS and MTS. In comparison, CTS and sex display discernable intra‐feature separation but SHAP values of smaller magnitudes, clustering about the centre axis and slightly skewed to the positive side.

**Figure 3:**
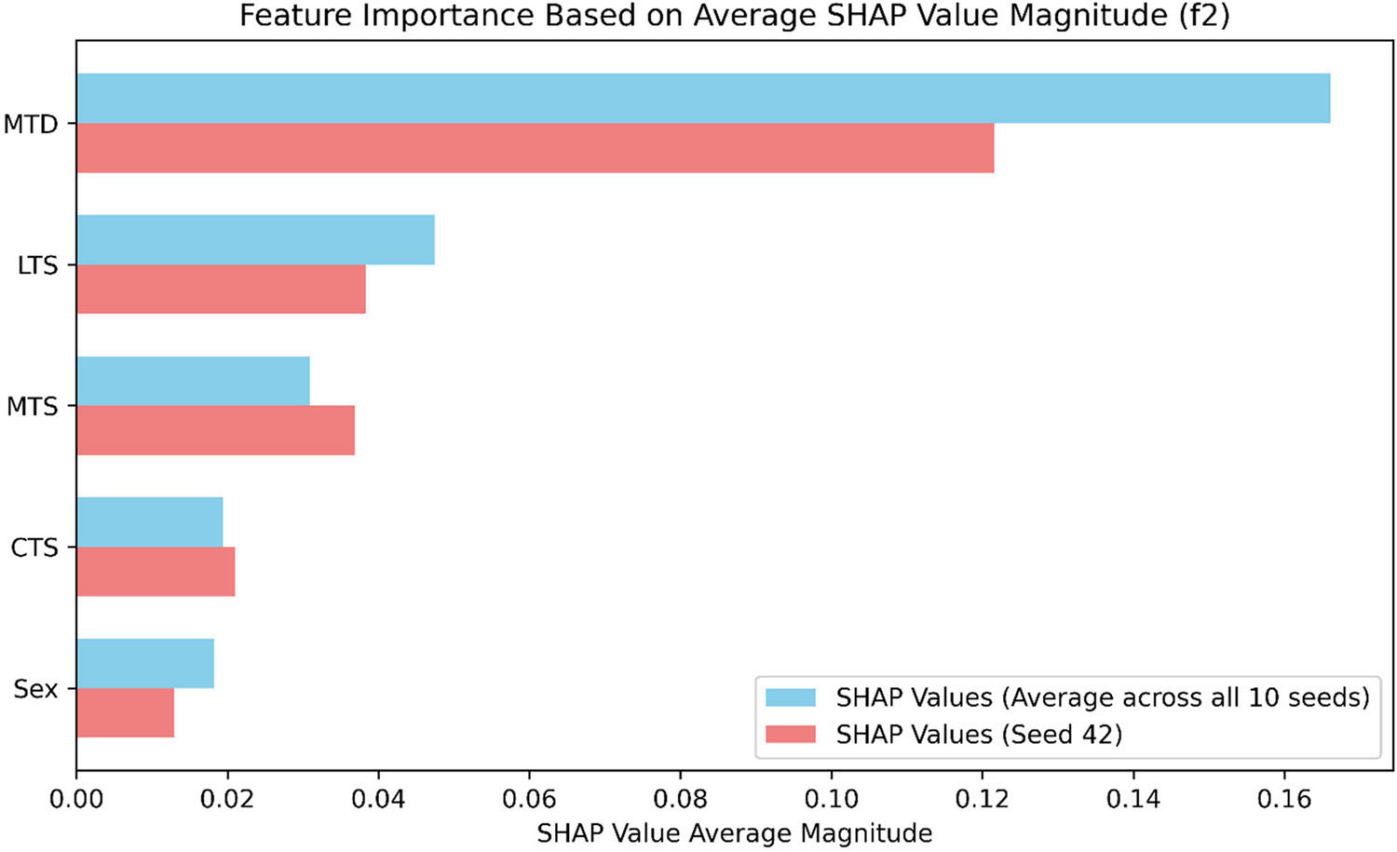
The SHapley Additive exPlanations (SHAP) summary plot showcases the averaged mean absolute SHAP values of different features collected from L2 weighted ensemble models across all 10 random seeds. The *Y*‐axis provides a ranking of tibial anatomical feature importance based on the mean absolute SHAP values. The *X*‐axis represents the contribution of tibial anatomical features to each example, manifested as mean absolute SHAP values.

Since the SHAP values obtained in Figure 2 are specific to the *default tested model*, they were compared against the average SHAP value obtained across all ten random seeds (Figure 3) for consistency. The feature importance rankings from the *default tested model* align with the averaged results across all seeds.

**Figure 4:**
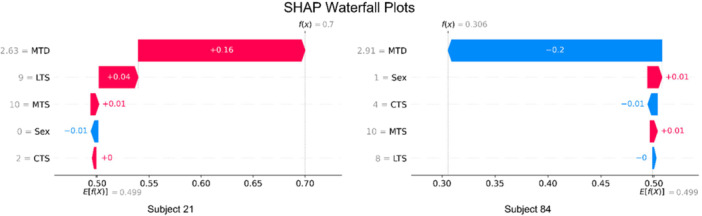
The representative SHapley Additive exPlanations (SHAP) waterfall plots showcase two (correct) predictions made by the *default tested model* on two different subjects. The subfigure on the left corresponds to model prediction of a lower likelihood of ACL injury, while the one on the right corresponds to a higher likelihood of ACL injury. The *y*‐axis provides the actual feature values measured from the individual subject in the order of their respective local SHAP values. *E*[*f*(*X*)] denotes the average prediction confidence score obtained from the train data, and *f*(*X*) denotes the final prediction confidence score for an individual subject. The blue and red elements capture the local feature importance, manifested by components of the difference between *E*[*f*(*X*)] and *f*(*X*).

In addition to global SHAP feature importance analysis, local SHAP explanation was enabled for both the *default tested model* and *full‐dataset model* to enhance interpretability. As depicted in Figure 4, the importance of each tibial anatomical feature is shown for individual predictions made by the *default tested model*, displaying both feature contribution and prediction confidence.

All experiments described in this section relied on the F2‐score as the validation target. For completeness, these experiments were repeated with both balanced accuracy and the F1‐score as validation targets. The recommended models are the *default tested model* and the *full‐dataset model*, but clinicians and researchers who prioritise balanced accuracy or the F1‐score may choose to use the corresponding models instead. All models will be available for demonstration or clinical use on the demo website (link in the Appendix).

## DISCUSSION

The most important finding of this study was that MTD, LTS and MTS can be jointly leveraged by ML models predict ACL injury with a satisfactory accuracy, providing interpretable, hypothesis‐generating insights. This study presents two versions of the top‐performing prognostic model for ACL injury prediction, leveraging various tibial anatomical features and sex. Both versions were tuned to optimise the F2‐score given the significant clinical risks associated with false negatives. The *default tested model* was selected via a comprehensive evaluation conducted across ten different random seeds. This model demonstrated a validation F2‐score of 73.60% as well as a test balanced accuracy of 95.44%, F1‐score of 95.24%, F2‐score of 98.04%, ROC AUC of 100%, precision of 90.91%, and recall of 100%. The *full‐dataset model* was trained on the entirety of the original dataset to exchange testability for maximum capabilities. This model achieved a validation F2‐score of 81.30%. SHAP established MTD, LTS and MTS as the top predictors for the *default tested model*.

To the best of the authors' knowledge, the study by Tamimi et al. represents the only prediction‐focused study among all literature that utilised ML as a tool for analysing the relationship between the different tibial slopes and ACL injury, distinguishing it from other studies that fail to differentiate between the tibial slopes [44]. This makes the model developed by Tamimi et al. the only appropriate, direct benchmark for comparison in the present study. Comparing the predictive power of the tested ML model presented by this study (the *default tested model*, balanced accuracy: 95.45%, accuracy: 95.24%, sensitivity: 100.0% and specificity: 90.91%) against that of Tamimi et al.‘s (accuracy: 92%, sensitivity: 92% and specificity: 92%), performance was highly comparable. Notably, both models yielded optimistic estimates due to favourable data splits, as indicated by the discrepancy between validation and test scores. Hence, both models' predictive performance on unseen independent data is anticipated to be closer to their respective validation results. The resemblance in predictive performance across the two studies signifies that the use of state‐of‐the‐art models in this study does not yield meaningful improvements in predictive capabilities over Tamimi et al.'s Gaussian naïve Bayes model, despite their specialised design to tackle small tabular datasets and account for nonlinear inter‐feature interactions. This observation suggests the predictive performance in this setting is data‐constrained rather than model‐constrained. Nonetheless, the current study validates the predictive utility of the included tibial anatomical parameters and provides model‐level interpretability via SHAP analysis, which has been widely adopted in recent ML studies to explain model predictions on ACL injury and post‐ACLR outcomes [20, 26, 28, 43, 45]. This offers deeper insights into the decision mechanisms in the provided model, which was not explored by Tamimi et al. [44].

The validation‐test discrepancy observed across the two studies highlights the limitations in reproducibility present in both studies arising from the use of small datasets. This necessitates and justifies the development of the *full‐dataset model*, which is better constrained by data and expected to be more stable in deployed settings in comparison to the *default tested model*. Further, it should be emphasised that this study does not claim superior model performance or infer potential risk factors based on evaluation of the full‐dataset model on any portion of the data. Rather, the *full‐dataset model* is proposed solely for potential future deployment, where training on the complete dataset may improve robustness and generalisability. Though not used for performance benchmarking, the *full‐dataset model* was regularised and validated to reduce the risk of overfitting to training data. Interestingly, the *full‐dataset model* produced local feature explanations consistent with those of the *default tested model*. All rigorous model evaluation and benchmarking reported in this study were conducted using the *default tested model* which strictly adhered to the established best practices for ML practice.

Globally, the feature explanations obtained from the *default tested model* concurred with the feature explanations from the ten‐seed aggregation, both concluding that feature importance is ranked in the order of MTD, LTS, MTS, CTS and sex, despite slight differences in numerical values. Further, a closer investigation of the SHAP beeswarm plot in Figure 2 suggests that decreased MTD (blue), increased LTS (red) and increased MTS (red) are top predictors contributing to model predictions associated with high likelihood of ACL injury. Notably, SHAP values describe how the model distributes importance across input variables, and do not establish causation or mechanistically identify biological risk factors. The global feature explanation reflects the *default tested model*'s internal decision patterns within this dataset and should not be interpreted as evidence of true anatomical risk or mechanistic pathways. Rather, SHAP results serve as hypothesis‐generating insights that outline potential risk factors that may help guide future biomechanical studies.

The findings regarding global feature importance from the *default tested model* mostly align with existing literature on tibial anatomical risk factors. For instance, increased LTS and MTS have been identified by review studies as important risk factors for non‐contact ACL injury, influencing ACL force distribution, translation on the lateral tibial plateau and pivot shifts to the medial tibial plateau [3, 47, 52]. Similarly, Pfeifer et al., [35] has summarised across studies, that both increased LTS and MTS are widely regarded as risk factors, particularly in females, where they may exacerbate injury likelihood. Furthermore, increased MTS and LTS have been established by a review study as risk factors for ACL injury, with the ratio of MTS to LTS showing a negative correlation with knee abduction, consistent with current understandings of ACL injury mechanisms [8]. Despite slight differences in results due to geographical variation, it is becoming increasingly evident that LTS and MTS are crucial for predicting ACL injury, and that slope‐reducing osteotomy should be further investigated as a potential prevention mechanism [52]. Relative to other previously discussed tibial slopes, CTS is identified as a weak predictor of ACL injury. This finding concurs with the limited number of available literature on CTS, which concluded that CTS was not a risk factor [16, 46].

Unlike tibial slopes, the MTD is much more complicated. On one hand, studies like [16, 36] identified MTD as a risk factor. On the other hand, some works failed to observe statistical significance in the difference between MTD across groups [18, 33]. This likely stems from the fact that MTD is not an independent risk factor, which makes it more difficult to identify using traditional statistical tests. Biomechanically, LTS and MTS directly and independently contribute to the forces exerted on the tibia and the ACL, qualifying them as independent risk factors. MTD, on the contrary, does not have a discernible effect until combined with a steep tibial slope [35]. The depth of the tibial plateau merely determines the geometric constraints on knee kinematics, and is only problematic when combined with increased tibial slopes; without a larger MTD to interfere with the translation induced by increased LTS and MTS, the posterior translation of the tibia is unrestrained, placing increased strain on the ACL and elevating the risk of rupture [40]. Therefore, MTD is a dependent but synergistically critical predictor of ACL injury, yielding the highest global SHAP value in this study.

Sex being elucidated as the least predictive feature was the most surprising result for the authors, given that sex is commonly recognised as a risk factor for ACL injury due to the higher incidence observed among females [5]. This unanticipated result is attributed to sex being correlated with other variables and features included in this study. Some works, for example, categorise sex as an intermediate risk factor, suggesting its association with ACL injury is mediated by tibial slopes, MTD, and related dynamic instability in the tibiofemoral region [9, 15, 19]. According to these studies, women were observed to have increased LTS and MTS with decreased MTD, which aligns with Table 3. It is likely there exists a sex‐correlated non‐linear interaction among MTD, LTS and MTS that captures the biomechanical mechanisms of ACL injury, and that the interaction has been learnt by the ML models from the data provided. As a result, they attend more to the tibial anatomical features which are semantically richer, discarding sex as a mediated and redundant feature.

While clinical thresholds could be derived from the study, the authors argue against their use since they do not adequately capture the complex non‐linear interactions between different tibial anatomical features. The same numerical value of MTS, based on the authors' findings, can correspond to different injury outcomes, depending on the magnitude of the MTD and LTS. An example of this is showcased in Figure 4, where subjects #21 and #84 share the same MTS values, but are assigned opposite predicted outcomes (and ground truth labels). Instead of relying on clinical thresholds, the integration of explainable, non‐linear ML models offers superior performance. From a clinical perspective, the present findings suggest that tibial anatomical parameters such as MTD, LTS and MTS may serve as informative features for ACL injury prediction in clinically relevant settings.

While this study highlights the necessity of ML models, limitations are acknowledged. First, the dataset utilised is considerably smaller than that required for the problem to be Probably Approximately Correct learnable [17]. In the absence of this property, a significant risk exists that the data sampled from the underlying distribution may be unrepresentative, rendering the model prone to overfitting. Consequently, the model's capacity to generalise effectively to unseen data and to draw robust conclusions regarding the predictive power of features is compromised. The power analysis performed indicates that the dataset only provides adequate power to detect moderate to strong associations, and cannot differentiate weak associations of odds ratio less than 1.7 from noise in the data. In addition, considering that ML models can capture non‐linear interaction effects beyond the scope of classical power analysis, the current findings should be interpreted as exploratory, requiring larger cohorts for external validation. To best mitigate this, multi‐layer stacking with k‐fold bagging was used as means of variance reduction. Ideally, increasing dataset size would serve as a superior regularisation strategy that could result in better convergence of model predictions and feature explanation, and eliminate the need for a full‐dataset model that cannot be evaluated. However, this is not always feasible for ML studies in orthopaedics due to cost and complexity constraints associated with large‐scale data collection and annotation.

Some other limitations include a restricted set of anatomical features and the absence of standardised techniques for measuring tibial slopes and depths across the field [49]. Specifically, the ML models incorporated only a subset of biomechanical features derivable from MRI scans, such as tibial slopes and depth, while omitting other relevant factors like cartilage thickness, femoral notch width and meniscal slopes due to data unavailability. This limited scope encourages the exploration of connections between tibial slopes/depth and ACL injury, but also hinders the models' ability to fully capture the complex interactions underlying the orthopaedic condition. Time‐dependent features such as age and weight are also potentially important variables to complement the time‐invariant tibial anatomical features given that the development of ACL injury is time‐dependent. Furthermore, the lack of validated standards for generating anatomical measurements further constrains the potential of MRI data to inform accurate and generalisable ML predictions, as pointed out by Wordeman et al. [49].

Future studies could work on integrating tibial slope measurement models investigated in [24, 27, 34, 37, 50]. This could establish an end‐to‐end predictive framework that automates MRI feature extraction, minimising manual annotation and enabling real‐time predictions. Also, applying the same modelling techniques and evaluation procedures to larger, streamlined datasets broader feature coverage could improve model generalisation and mitigate the risk of overfitting, leading to more robust predictive performance and feature attribution. Finally, a systematic comparison of different tibial slope measurement techniques could be conducted to assess their relative robustness and reliability, informing the selection of techniques for future research efforts.

## CONCLUSION

In conclusion, this study presents two versions of state‐of‐the‐art prognostic models for predicting injury outcomes and identifying potential risk factors of ACL injury from tibial anatomical features and sex. The models demonstrate a satisfactory level of predictiveness. The models are available for access on the demo website. The global SHAP analysis results align with existing literature, identifying decreased MTD, increased LTS and increased MTS as the most contributive tibial plateau morphological features. Overall, the models developed through this study laid the foundation for future research and can be further integrated with existing ACL registries to improve generalisability or combined with other studies to accomplish more complex tasks.

## AUTHOR CONTRIBUTIONS

Cheng‐Hao Kao developed the ML pipeline, conducted subsequent analysis, and wrote the manuscript. Javad Hashemi and James Slauterbeck collected the original data, reviewed and revised the manuscript. Naveen Chandrashekar as the principal investigator, guided the study, co‐wrote the manuscript and provided final critic review.

## CONFLICT OF INTEREST STATEMENT

The authors declare no conflicts of interest.

## ETHICS STATEMENT

The dataset used was previously published and is publicly available. The ethics board has confirmed that ethical approval is not required for this study.

## Data Availability

Data are publicly available.

## Appendix

Demo website link: https://bluetr981.github.io/ACL-Injury-Website/.
